# Supplementary material for: Upgrading of Biobased Glycerol to Glycerol Carbonate as a Tool to Reduce the CO2 Emissions of the Biodiesel Fuel Life Cycle
Source: Bioengineering (Basel). 2022 Dec 6;9(12):778. doi: 10.3390/bioengineering9120778 (PMC9774724; doi:10.3390/bioengineering9120778)
Supplement: Supplementary file 1 [file bioengineering-09-00778-s001.zip › bioengineering-2050685-supplementary.pdf]

bioengineering

MDPI

Article

Upgrading of Biobased Glycerol to Glycerol Carbonate as a Tool to Reduce the CO_2 Emissions of the Biodiesel Fuel Life Cycle

Biagio Anderlini [1], Alberto Ughetti [1], Emma Cristoni [1], Luca Forti [2], Luca Rigamonti [1,3,4] and Fabrizio Roncaglia [1,3,4,*]

[1] Department of Chemical and Geological Sciences, University of Modena and Reggio Emilia, Via G. Campi 103, 41125 Modena, Italy
[2] Department of Life Sciences, University of Modena and Reggio Emilia, Via G. Campi 103, 41125 Modena, Italy
[3] Interdepartmental Centre H_2-MORE, University of Modena and Reggio Emilia, Via Università 4, 41121 Modena, Italy
[4] INSTM Research Unit of Modena, Via G. Campi 103, 41125 Modena, Italy
* Correspondence: fabrizio.roncaglia@unimore.it

Citation: Anderlini, B.; Ughetti, A.; Cristoni, E.; Forti, L.; Rigamonti, L.; Roncaglia, F. Upgrading of Biobased Glycerol to Glycerol Carbonate as a Tool to Reduce the CO_2 Emissions of the Biodiesel Fuel Life Cycle. *Bioengineering* **2022**, *9*, 778. https://doi.org/10.3390/bioengineering9120778

Academic Editors: Indra Neel Pulidindi, Aharon Gedanken, Giorgos Markou

Received: 7 November 2022
Accepted: 25 November 2022
Published: 6 December 2022

Publisher's Note: MDPI stays neutral with regard to jurisdictional claims in published maps and institutional affiliations.

Copyright: © 2022 by the authors. Licensee MDPI, Basel, Switzerland. This article is an open access article distributed under the terms and conditions of the Creative Commons Attribution (CC BY) license (https://creativecommons.org/licenses/by/4.0/).

SUPPLEMENTARY

Products characterization.

After preliminary evaluation, Gly-urea mixtures were processed within the DoE domain, that is: reactor temperature from 175 to 195 °C, recirculation time from 90 to 210 min, urea:Gly MR from 1.2 to 1.8. After solvent extraction (EtOAc:Et_2O 4:1) and drying, a colorless oil was obtained. This raw material was directly analyzed with ^1H NMR spectroscopy and two main groups of signals were detected:

- a set of signals (Figure S1) was assigned to GC;

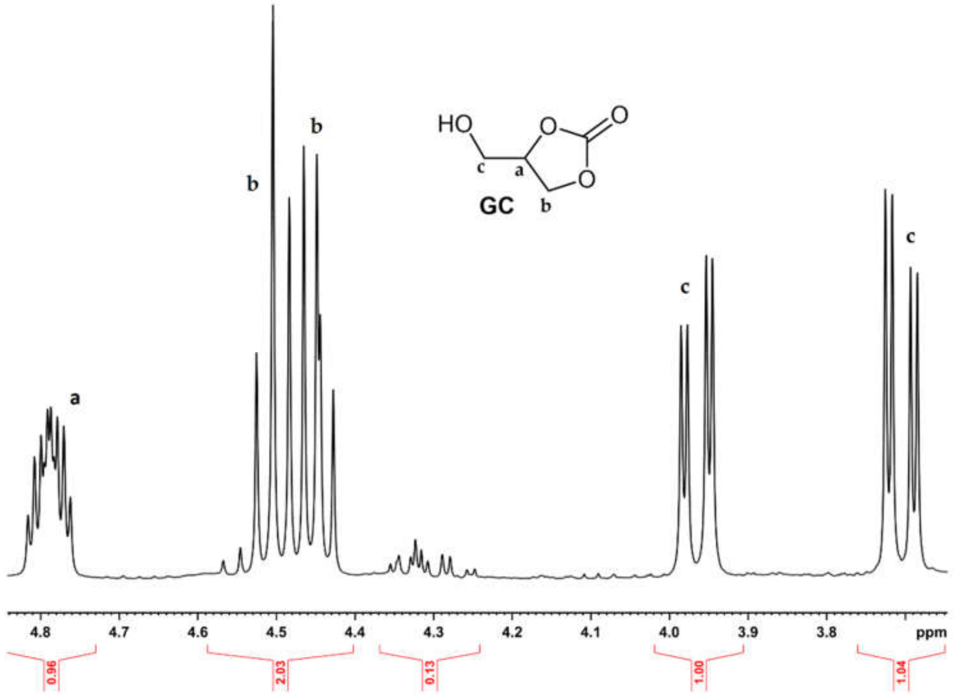

Figure S1. Typical ^{1}H-NMR spectra ($CDCl_3$, 400 MHz) of a GC-rich raw mixture.

- a second set of signals was assigned to DGTC. DGTC was always detected as contaminant of the main product (GC) and its identity was gathered from the literature [94-96]. In Figure S2 is shown a typical ^{1}H NMR spectra of a DGTC-rich raw mixture.

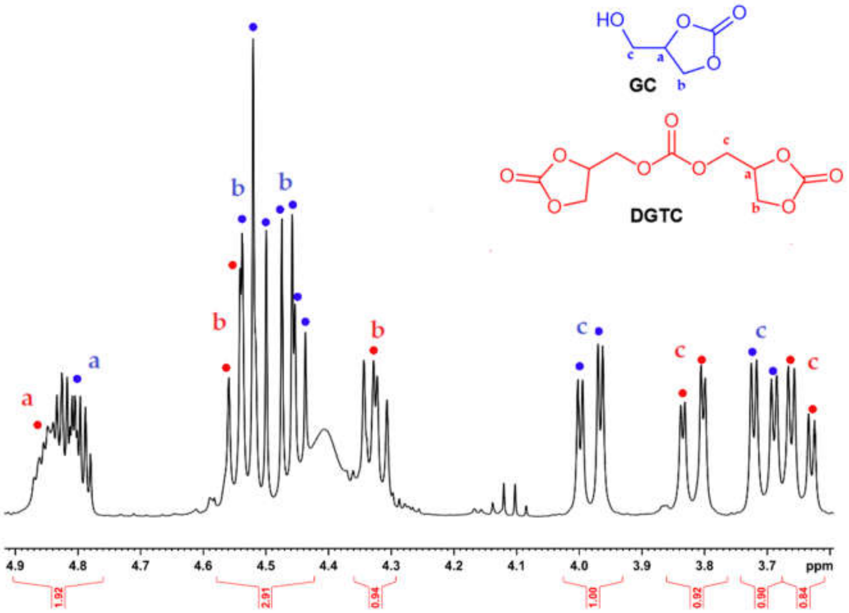

Figure S2. Typical ^{1}H NMR spectra ($CDCl_3$, 400 MHz) of a DGTC-rich raw mixture.

As DGTC was the only recurring and largely predominant detected side-product, we approximated the raw (extracted) reaction mixture as a binary mixture. Given that, GC molar selectivity was calculated from the integrated area of isolated $-CH-C\underline{H}_2-OH$ signals (**c** in Figg S1 and S2) of the desired product (GC, dd at δ 3.99 ppm, 1H) and the contaminant (DGTC, dd at δ 3.82 ppm, 2H), as follows:

$$\text{GC Sel \%} = \frac{100 \cdot \text{Area}\ (\delta\ 3.99\ \text{ppm})}{\text{Area}\ (\delta\ 3.99\ \text{ppm}) + (\text{Area}\ (\delta\ 3.82\ \text{ppm})\ /2)}$$

Figure S2 depicts the ^{1}H NMR spectra of the raw mixture obtained after solvent extraction from experiment n. 3 (Table 2). In this case, the calculated value for GC selectivity% equals to 68 (= 100·(1.00/1.00+(0.92/2))).
